# Emergency department visits and associated factors among people with dementia residing in nursing homes in Taiwan: a one-year cohort study

**DOI:** 10.1186/s12877-023-04221-5

**Published:** 2023-08-21

**Authors:** Jiun-Yi Wang, Yu-Wan Yang, Chien-Hui Liu, Kun-Chia Chang, Yi-Ting Lin, Chih-Ching Liu

**Affiliations:** 1 Department of Healthcare Administration, College of Medical and Health Science, Asia University, 500, Lioufeng Rd, Wufeng, Taichung, 41354 Taiwan; 2 Department of Medical Research, China Medical University Hospital, China Medical University, Taichung, Taiwan; 3 Department of Neurology, China Medical University Hospital, Taichung, Taiwan; 4 College of Medicine, China Medical University, Taichung, Taiwan; 5 Institute of Biomedical Informatics, National Yang Ming Chiao Tung University, Hsinchu, Taiwan; 6 Division of Emergency Medical Service, New Taipei City Fire Department, New Taipei, Taiwan; 7 Jianan Psychiatric Center, Ministry of Health and Welfare, Tainan, Taiwan; 8 Department of Psychiatry, National Cheng Kung University Hospital, College of Medicine, National Cheng Kung University, Tainan, Taiwan

**Keywords:** Nursing home, Dementia, Emergency department utilization, Retrospective cohort study, Andersen model

## Abstract

**Background:**

Residing in a nursing home (NH) may increase emergency department (ED) utilization in patients with dementia; however, evidence regarding the status of and predictors for ED utilization of NH residents with dementia remains unclear, especially in Asia. This study aimed to assess the incidence density of ED visits and associated factors for the risk of ED utilization among NH residents with dementia.

**Methods:**

This one-year cohort study followed 6595 NH residents with dementia aged ≧ 40 years from Taiwan’s National Health Insurance Research Database between 2012 and 2014. The Andersen-Gill extension of Cox regression analysis with death as a competing risk was applied to investigate the association of the risk of all causes and the most common causes of ED utilization with the predisposing, enabling, and need factors as defined by the Andersen model.

**Results:**

All participants encountered 9254 emergency visits in the 5371.49 person-years observed, representing incidence densities of ED visits of 1722.80 per 1000 person-years. Among them, respiratory disease was the most common cause of ED visits. The significant predictors for the risk of all-cause and respiratory-cause ED visits included: (1) predisposing factors (i.e., age and gender); (2) enabling factors (i.e., regional variables); and (3) need factors (i.e., prolonged ventilator dependence and comorbidity status).

**Conclusions:**

Predisposing, enabling, and need factors could influence ED visits among studies patients. NH providers should consider these factors to develop strategies for reducing ED utilization.

## Background

Dementia currently affects nearly 55 million people globally, and there are approximately 10 million new cases annually [1]. In addition to being the most common neurodegenerative disorder [2], it is the leading cause of dependency and disability among elderly people worldwide [3]. Moreover, it is a progressive disease that may cause mild to severe cognitive impairment and impairment of self-care ability in daily life. Thus, over their disease duration, they may have the need for support from formal or informal caregivers. Although the priority for people with dementia is aging in place at home [4], many of them transition to live in nursing homes (NH) to fulfill their increased needs for care and supervision [5].

Patients with dementia, whether they live at home or in NH, may seek emergency department (ED) care when experiencing acute medical conditions [6, 7]. Although providing specialized care in the ED is helpful for patients with dementia, the overstimulating and unfamiliar environment from ED such as fast pace, high background noise, and many unfamiliar people may lead to their poor health outcomes (e.g., increased risk of aggression and confusion) [8] and increase their medical burden to health care system [9]. Moreover, dementia is a significant risk factor for increased hospitalization from the ED and mortality after ED visits [6, 10]. Despite that, many ED visits among these patients are considered to be preventable or unnecessary [11]. A better understanding of the causes or predictors of ED visits will help health-care providers develop strategies for decreasing ED utilization among these patients.

The predictive factors of ED utilization among people with dementia have been widely studied [6, 9, 10, 12, 13]. It is not surprising that living in NH was one of the significant independent factors of ED visits among people with dementia [9] because those living in NH were older, with more comorbidities, and with more functional impairment than those not living in NH [6]. Although over one-half of NH residents with dementia experience at least once ED visit within a year [6], very limited studies have been conducted to explore predictive factors for all-cause and specific-cause ED visits for NH residents with dementia, especially in the newly admitted NH residents. Additionally, no study has considered death as a competing risk to investigate the predictive factors of ED utilization among NH residents with dementia [6, 9, 10, 12, 13].

Considering the aforementioned research gap, we aimed to estimate the incidence density (ID) of and predictors of the risk of all-cause and specific-cause ED visits among NH residents with dementia in Taiwan by conducting a retrospective one-year follow-up cohort study with a large sample size and taking death as a competing risk. Additionally, given that Anderson’s Behavior Model of Health Services is the mostly widely used theoretical framework to predict and explain the use of health-care services [14], we used the model to further explore significant predictors for risk of all-cause and common specific-cause of ED visits.

## Methods

### Data source

The analyzed data in this study were retrieved from Taiwan’s National Health Insurance Research Database (NHIRD), which is maintained by the National Health Insurance Administration (NHIA), Ministry of Health and Welfare, Taiwan. Since 1995, the National Health Insurance (NHI) program has been implemented for Taiwanese residents [15]. In December 2002, more than 99% of the total Taiwanese population were enrolled into the NHI program, and 97% of hospitals and 90% of clinics across the nation are contracted by the NHIA [15]. The NHIRD includes registration and claims files from all hospitals and clinics. To ensure the accuracy of the claims files in the NHIRD, the NHIA performs an expert review on a random sample of claims data every quarter, and false diagnosis reports are given a severe penalty from the NHIA [16]. Thus, information obtained from the NHIRD is considered to be complete and accurate.

### Study design, setting, and participants

This is a retrospective one-year follow-up cohort study. The study participants were considered to have dementia if (1) they had three ambulatory claims with dementia-related diagnosis codes (International Classification of Diseases, Ninth Revision, Clinical Modification [ICD-9-CM] codes of 290, 294, 331, and 046.1) from 2011 to 2014, and (2) their first and last outpatient visits had to be at least 90 days apart to avoid the accidental inclusion of miscoded patients [17].

The data of these patients with dementia aged ≥ 40 years (n = 359,854) were further linked with the ambulatory claims between 2011 and 2014 to identify those who received home-based care in NH (No of case type = A6) during these periods (n = 12,466). This study only focused on these patients who received home-care service in NH because the NH information of NHIRD available was those claims made in the home care services data. Given the median survival time of NH residents with dementia was about one year [18], patients were considered newly admitted NH residents with dementia who did not have any record of admission to NH during the washout period (i.e., the year 2011). Subsequently, the study cohort consisted of a total of 6595 patients with dementia who received home-based care in NH for the first time between 2012 and 2014. The index date of the study cohort was the date when they received home-based care for the first time in NH.

### Predictive factors

In this study, Andersen’s Behavior Model of Health Services Use was used to identify predictors of ED visits among NH residents with dementia. Candidate predictors of health service utilization were classified into three categories: predisposing, enabling, and need factors [14]. Age at the index date and gender were included as predisposing factors because these factors may be related to predisposition toward the use of health service [14]. The geographic location and the level of urbanization [19] in Taiwan were identified from the Taiwan NHI insured’s data (i.e., the locations of the group insurance applicants) at the index date. To control diverse administrative characteristics, the counties/cities of study participants’ insured areas were grouped into six geographic areas (Taipei, northern, central, southern, Kao-Ping, and Eastern divisions) according to the administrative districts of the NHIA [20, 21]. Given that sparse participants in offshore islands (Kinmen and Lienchiang Counties), participants in these offshore islands were not included in the study. Additionally, township areas were further categorized based on the levels of township urbanization, ranging from 1(lowest) to 7 (highest). The levels of township urbanization were determined using indicators such as population density, proportion of residents with a college education or higher, proportion of people older than 65 years, proportion of the agricultural workforce, and number of physicians per 100,000 population [19]. To maintain adequate statistical power, levels of township urbanization were re-categorized from 7 to 3 levels: urban (levels 1–2), suburban (levels 3–4), and rural (levels 5–7). Moreover, geographic locations and urbanization levels were used as proxy indicators to account for the possible accessibility and availability of medical care [22–26], and were classified as enabling factors, which may enable or impede their use of health service [14]. Need factors is based on individuals’ needs for health care [14]. It involved catastrophic illness (PART_NO = 001), prolonged mechanical ventilator dependence (ICD-9-CM = 51885), Charlson’s score [27], and Charlson’s specific comorbidities [27] such as stroke (ICD-9-CM = 430–438), chronic obstructive pulmonary disease (COPD) (ICD-9-CM = 490–496), peptic ulcer (ICD-9-CM = 531–534), cancer (ICD-9-CM = 140–208), congestive heart failure (ICD-9-CM = 428), and diabetes-related complications (ICD-9-CM = 250.0, 250.1, 250.2, 250.3, 250.7) that may be related to ED utilization within one year before the index date [12, 13, 28]. The catastrophic illness (including 30 categories, as presented in a previous study [29]) were diagnosed by physicians following the NHIA-defined guidelines [30]. Patients who received the certifications for catastrophic illnesses are exempt from copayments when seeking medical services [30]. The Charlson’s score was calculated during one year before the index date, which is weighted summery measure of common comorbid conditions by utilizing the ICD-9-CM coding developed by Deyo for use on administrative databases [27]. Additionally, the Charlson’s specific comorbidities were identified in the analysis when patients had diagnostic codes of these comorbidities at least 3 times within one year in outpatient claims or one time in inpatient claims during one year before index date. Based on the frequency distribution of Charlson score in the study cohort, the Charlson score was categorized into four groups (1, 2, 3, ≧4).

### Outcome measures

The primary outcome measure was any ED visits over the study periods. The study periods were from the index date to death or the end of one-year follow-up time, which ever occurred first. By case type (No. = 2), information on visits to the hospital ED was extracted from the ambulatory claim files. In this study, we used ICD-9-CM codes to identify all-cause and specific-cause ED visits, such as respiratory, genitourinary, circulatory, endocrine, and mental disorders.

### Statistical analyses

The baseline characteristics of participants are presented as counts and percentages for categorical variables and as means and standard deviations (SD) for discrete or continuous variables. We identified the top 10 leading causes of ED visits using major ICD-9-CM diagnostic codes among those participants. Additionally, by diagnostic groups, the all cause and specific-cause incidence densities of ED visits were calculated as the number of ED utilization in study participants divided by the total person-years, yielding rates per 1000 person-years of observation. The ID of all-cause and specific-cause number of ED utilization was calculated using primary and secondary ICD-9-CM codes. The total person-years was calculated from the index date to the end of one-year follow-up time or death, whichever came first. Moreover, for multivariable analyses, we conducted the Andersen-Gill (AG) extension of Cox proportional hazard model with death as a competing risk to explore the associations between the risk of any all-cause and the most common specific-cause ED visits and potential independent variables [31–34]. This AG method (counting process) of modeling allows for the use of multiple ED visits events per participant (i.e., all the observed ED visits events) over time, while appropriately accounting for the correlation [31, 33, 34]. The rate of multiple ED visits events in the model is measured as the time between visits counted over the study period [31, 32]. The hazard ratios (HR) were calculated by comparing the rate of occurrence of multiple ED visits in participants with and without specific independent variables [31, 34], including predisposing factors, enabling factors, and need factors. A *p* value of < 0.05 was considered statistically significant. The SAS (Version 9.4; SAS Institute Inc., Cary, NC, USA) was used to perform all statistical analyses.

## Results

### Patient characteristics

Of the 6595 NH residents with dementia identified in this study, 3183 (48.33%) were male, with a mean age of 81.38 years. The distribution of the index year was similar among study participants. Additionally, they were more likely to be from urban areas such as Taipei, as well as from the central and southern regions. The proportions of their Charlson’s score of 1, 2, 3, ≧4 were 21.67%, 33.80%, 20.06%, and 24.47%, respectively. Stroke (63.23%) was the most prevalent comorbidity, followed by peptic ulcer and COPD. Moreover, 33.86% had a catastrophic illness, and 1.70% were prolonged mechanical ventilation-dependent patients (Table 1).

**Table 1:** Baseline characteristics of nursing homes residents with dementia

Variables^a^	n	%
Age (years)		
40–64	543	8.23
65–69	258	3.91
70–74	474	7.19
75–79	912	13.83
80–84	1445	21.91
85–89	1644	24.93
≧90	1319	20.00
Mean ± SD	81.38 ± 10.56	
Gender		
Male	3183	48.33
Female	3403	51.67
Index year		
2012	1999	30.31
2013	2196	33.30
2014	2400	36.39
Geographic area		
Taipei region	1695	25.75
Northern region	697	10.59
Central region	1609	24.43
Southern region	1557	23.64
Kao-Ping region	830	12.60
Eastern region	197	2.99
Urbanization		
Urban	2848	44.96
Suburban	2245	35.44
Rural	1241	19.60
Charlson’s score		
1	1429	21.67
2	2229	33.80
3	1323	20.06
≧ 4	1614	24.47
Mean ± SD	2.70 ± 1.61	
Comorbidities		
Stroke	4170	63.23
COPD	1885	28.58
Diabetes-related complications	108	1.64
Peptic ulcer	1922	29.14
Cancer	669	10.41
Congestive heart failure	1173	17.79
Catastrophic illness	2233	33.86
Prolonged mechanical ventilation-dependent	112	1.70
Total	6595	100.0

SD = standard deviation; COPD = chronic obstructive pulmonary disease

^**a**^Inconsistency between total population and population summed for individual variables was due to missing information

### Frequency and causes for emergency department visit

Overall, a total of 6595 study participants had 9254 ED visits in the follow-up period. Pneumonia, organism unspecified, was the most common cause of ED visits (14.8%), followed by fever, unspecified (10.5%) and urinary tract infection, site not specified (7.6%) (Table 2).

**Table 2:** Top 10 causes of emergency department visits

Rank	Emergency diagnosis (ICD-9-CM)	No. of emergency visit (n = 9254)	
		n	%
1	Pneumonia, organism unspecified (486)	1370	14.8
2	Fever, unspecified (780.6)	970	10.5
3	Urinary tract infection, site not specified (599.0)	706	7.6
4	Hearing loss (389)	413	4.5
5	Acute respiratory failure (518.81)	284	3.1
6	Hemorrhage of gastrointestinal tract, unspecified (578.9)	253	2.7
7	Other dyspnea and respiratory abnormalities (786.09)	202	2.2
8	Chronic kidney disease (585)	167	1.8
9	Chronic airway obstruction, not elsewhere classified (496)	147	1.6
10	Obstructive chronic bronchitis with acute exacerbation (491.21)	133	1.4

The ID of all-cause ED visits was 1722.80 per 1000 person-years. Respiratory disorders (770.74 per 1000 person-years) exhibited the highest ID of ED visits for different specific causes, followed by genitourinary (522.95 per 1000 person-years) and circulatory disorders (398.59 per 1000 person-years) (Table 3).

**Table 3:** Emergency department visit among residents with dementia in nursing homes

Diagnostic group (ICD-9-CM)	No. of emergency visit^a^	ID^b^	95%CI
All causes	9254	1722.80	1687.70-1757.90
Respiratory (460–519)	4140	770.74	747.26-794.21
Symptoms, signs and ill-defined conditions (780–799)	3745	697.20	674.87-719.53
Genitourinary (580–629)	2809	522.95	503.61-542.29
Circulation (390–459)	2141	398.59	381.70-415.47
Endocrine (240–279)	1488	277.02	262.94-291.09
Digestive (520–579)	1148	213.72	201.36-226.08
Infection (001-139)	1039	193.43	181.67-205.19
Mental (290–319)	804	149.68	139.33-160.03
Injury and poisoning (800–999 & E800-E999)	637	118.59	109.38–127.80
Neoplasm (140–239)	567	105.56	96.87-114.25
Nerve (320–389)	441	82.10	74.44–89.76
Skin (680–709)	353	65.72	58.86–72.57
Blood (280–289)	303	56.41	50.06–62.76
Musculoskeletal (710–739)	108	20.11	16.31–23.90
Congenital anomalies (740–759)	6	1.12	0.22–2.01
Other factors influencing health status and contact with health services (V00-V82)	3	0.56	-0.07-1.19

ID = incidence density; CI = confidence interval

^a^ Total person-years: 5371.49 person-year

^**b**^ Per 1000 person-years

### Predictive factors for all-cause and respiratory-cause ED visits

Compared with participants aged 40–64 years, those aged 90 years and more had a very high adjusted hazard ratio (AHR) of all-cause (1.55, 95% confidence interval [CI] = 1.29, 1.86) or respiratory-cause (2.23, 95% CI = 1.74, 2.86) ED visits. Additionally, male patients with dementia had a slightly higher AHR of all-cause (1.20, 95% CI = 1.11, 1.30) or respiratory-cause (1.63, 95% CI = 1.46, 1.81) ED visits than their female counterparts.

Among the enabling factors such as geographic location, the Taipei region (AHR = 1.29, 95% CI = 1.02, 1.62), the central region (AHR = 1.34, 95% CI = 1.07, 1.68), and rural areas (AHR = 1.19, 95% CI = 1.06, 1.33) were significant predictors of the higher risk of all-cause ED visits in the adjusted model. By contrast, patients who lived in the southern region (AHR = 0.76, 95% CI = 0.60, 0.97) and the Kao-ping region (AHR = 0.71, 95% CI = 0.55, 0.92) were less likely to use ED services. The association of these enabling factors with the risk of respiratory-cause ED visits was also similar across geographic locations, such as the Taipei region (AHR = 1.74, 95% CI = 1.25, 2.43) and the central region (AHR = 2.09, 95% CI = 1.51, 2.91).

In terms of need factors, patients with a history of prolonged mechanical ventilator dependence exhibited significantly increased the risk of all-cause (AHR = 1.73, 95% CI = 1.33, 2.23) and respiratory-cause (AHR = 1.73, 95% CI = 1.23, 2.43) ED use. Among Charlson’s score and specific comorbidity factors, only a higher Charlson’s score was significant for predicting the risk of all-cause ED visits, with the highest Charlson’s score of ≥ 4 being the most likely to increase the risk of all-cause ED utilization (AHR = 1.32, 95% CI = 1.14, 1.52), whereas cancer (AHR = 1.25, 95% CI = 1.09, 1.43) and COPD (AHR = 1.37, 95% CI = 1.22, 1.53) were significantly related to the risk of all-cause and respiratory-cause ED visits, respectively **(**Table 4**)**.

**Table 4:** Hazard ratio of emergency department visits for nursing homes residents with dementia

Variables	All-cause			Respiratory-cause		
	AHR^a^	95% CI	*P*	AHR^a^	95% CI	*P*
Predisposing factors						
Age (years)						
40–64	1.00			1.00		
65–69	1.58^*^	1.22–2.04	< 0.001	1.69^*^	1.17-2.44	0.005
70–74	1.47^*^	1.19–1.81	< 0.001	1.82^*^	1.36-2.44	< 0.001
75–79	1.53^*^	1.26–1.86	< 0.001	1.92^*^	1.47–2.50	< 0.001
80–84	1.46^*^	1.22–1.75	< 0.001	1.90^*^	1.48–2.44	< 0.001
85–89	1.50^*^	1.25–1.79	< 0.001	2.11^*^	1.65-2.69	< 0.001
≧90	1.55^*^	1.29–1.86	< 0.001	2.23^*^	1.7–2.86	< 0.001
Gender						
Male	1.20^*^	1.11-1.30	< 0.001	1.63^*^	1.46–1.81	< 0.001
Female	1.00			1.00		
**Enabling factors**						
Geographic area						
Taipei region	1.29^*^	1.02–1.62	0.038	1.74^*^	1.25-2.43	0.001
Northern region	1.03	0.80–1.32	0.831	1.38	0.97–1.96	0.075
Central region	1.34^*^	1.07–1.68	0.011	2.09^*^	1.51-2.91	< 0.001
Southern region	0.76^*^	0.60-0.97	0.024	1.03	0.74–1.45	0.856
Kao-ping region	0.71^*^	0.55–0.92	0.008	0.80	0.55–1.15	0.228
Eastern region	1.00			1.00		
Urbanization						
Urban	1.00			1.00		
Suburban	1.07	0.98–1.17	0.115	1.01	0.90–1.14	0.860
Rural	1.19^*^	1.06–1.33	0.004	1.11	0.94–1.31	0.214
**Need factors**						
Charlson’s score						
1	1.00			1.00		
2	1.15^*^	1.02–1.29	0.020	1.16	0.99–1.36	0.069
3	1.17^*^	1.03–1.34	0.017	1.09	0.91–1.30	0.350
≧ 4	1.32^*^	1.14–1.52	< 0.001	1.07	0.88–1.30	0.493
Comorbidities						
Stroke	1.01	0.93–1.11	0.756	1.12	1.00-1.26	0.055
COPD	1.03	0.95–1.12	0.479	1.37^*^	1.22–1.53	< 0.001
Diabetes-related complications	0.84	0.65–1.08	0.165	0.77	0.51–1.17	0.216
Peptic ulcer	0.99	0.91–1.08	0.834	0.96	0.86–1.08	0.488
Cancer	1.25^*^	1.09–1.43	0.001	1.19	0.99-1.43	0.065
Congestive heart failure	1.06	0.96–1.17	0.236	1.07	0.94–1.23	0.290
Catastrophic illness	0.99	0.91–1.08	0.852	1.03	0.92–1.16	0.578
Prolonged mechanical ventilator dependence	1.73^*^	1.33–2.23	< 0.001	1.73^*^	1.23–2.43	0.002

AHR = adjusted hazard ratio; CI = confidence interval; COPD = chronic obstructive pulmonary disease

^**a**^ Based on extensions of the Cox regression models (Andersen-Gill models) with death as a competing risk, adjusted for age, gender, geographic area, urbanization, Charlson’s score, comorbidities, catastrophic illness, and prolonged mechanical ventilator dependence

## Discussion

### Main findings

To the best of our knowledge, this is the first study to identify the predictive factors for the risk of all-cause and specific-cause ED visits among NH residents with dementia using Anderson model. Our findings revealed that respiratory disease was the most common cause of ED visits, especially for pneumonia. Additionally, the risk of all-cause ED visits was strongly associated with predisposing factors (age and gender), enabling factors (geographic location and urbanization level), and need factors (Charlson’s score, cancer, and prolonged mechanical ventilator dependence). Similar findings were shown in respiratory-cause ED visits, with the exception of the urbanization level, Charlson’s score, and cancer. Moreover, COPD was identified as a predictive factor for the risk of respiratory-cause ED visits.

### Emergency department utilization among nursing home residents with dementia

In previous studies, pneumonia, urinary tract infection, and congestive heart failure were the top three reasons for ED visits among older people living in long-term care institutions [35, 36]. This is consistent with our findings. Thus, reasons for these findings should be explored. First, previous studies found that more than 60% of NH residents with dementia had a dysphagia problem [37] likely the result of age-related functional decline and neuropathy [38]. This condition may increase respiratory-cause ED utilization among people with dementia because dysphagia is a leading cause of aspiration pneumonia [36, 39]. Second, some of our study participants were prolonged mechanical ventilation-dependent patients. These patients have poor respiratory function that may predispose pneumonia. Additionally, if their respiratory equipment was contaminated by bacteria or viruses, it might also cause pneumonia among these patients [40]. Moreover, our study showed that most of the NH residents with dementia were comorbid with stroke (63.23%) and COPD (28.58%). Previous studies have revealed that NH residents with stroke are less inclined to receive a pneumococcal vaccine [41]. Furthermore, patients with COPD have chronic airway infections [42]. These conditions might also contribute to the increased risk of pneumonia [41, 42] and the need for ED services [35, 36].

Regarding the explanations for ED utilizations related to urinary tract infection, we suspect that some NH residents were likely to have urinary catheter indwelling [43], which can increase the risk of urinary tract infection [44] and result in ED visits [35]. However, the true reasons for the increase the ED visits related to urinary tract infection in NH residents with dementia remains unclear and warrants further investigation. Moreover, NH residents with dementia comorbid with pneumonia or urinary tract infection may further increase the risk of sepsis [45], which could further increase the risks of hypotension [46] and heart failure [47] and thus the need of ED care [36]. Overall, these findings suggest that identifying and controlling respiratory- or urinary-related infection causes, as well as implementing interventions to protect NH residents with dementia from infections, can reduce the risk of ED visits.

### Association of predisposing, enabling, and needing factors with all-cause and respiratory-cause emergency department visits

According to our findings, the risk of all-cause and respiratory-cause ED visits could be predicted by predisposing factors (e.g., age and gender), and the risk of all-cause and respiratory-cause ED visits would increase with age. Our results may be explained by the following. First, residents with older age were more prevalent in dysphagia [48] and then may increase the risk of aspiration pneumonia among these patients [39]. Second, male patients are more likely to have poor immune systems than female patients [49].

In terms of the association between enabling factors and the risk of ED visits, we found that our study participants from Taipei and the central region were more likely to have a higher risk of all-cause and respiratory-cause ED visits than those from the eastern region. This might be because more majority of health-care resources and lower distance of transportation in accessing healthcare are allocated to the most northern region and central region [22, 23]. Thus, we suspected that NH professionals from Taipei and the central region would still refer patients in NH to ED even if patients’ conditions were not urgent because ED services are more accessible. Furthermore, our study found those from rural areas tended to visit the ED more frequently than those from urban areas. This finding could be attributed to the limited access to health-care services in the eastern region and rural areas [22, 24], which could worsen their medical conditions and increase their need for ED services [24].

In terms of the relationships between need factors and the risk of ED visits among NH residents with dementia, patients having cancer or higher Charlson’s scores were more likely to have all-cause ED utilization. The difference might be because patients having cancer or a higher Charlson’s score would have more comorbidities with higher severity [27, 50], leading to worsening of their health status and increasing the likelihood of ED service utilization [13]. However, the Charlson’s score was not a significant predictor of respiratory-cause ED visits. Respiratory-cause ED visits were still higher in those with specific comorbidities, such as COPD, and one possible explanation is that, as mentioned in our study, COPD are associated with an increased risk of the most common causes (i.e., pneumonia) of ED visits [42].

### Strengths and limitations

This study has several strengths. First, this study used data from the NHIRD, which provides a large sample size and sufficient of cohort, thereby providing higher statistical power. Second, this study provides more accurate estimates of the risk of ED visits because a multivariable extended Cox regression model using the AG method was used to account for the competing risk of death. Additionally, the use of the risk of ED visits rather than the occurrence (yes or no) of ED visits, which provided comprehensive insights into emergency utilization. In contrast, some limitations of this study should be mentioned. First, due to the limited NH information available from secondary data set, our study only included dementia patients who received home care services from NH covered by the NHI program rather than all dementia patients who resided in NH. In Taiwan, these patients are more likely to have higher severity disability and lower socioeconomic status than those without receiving home care services covered by the NHI program [51]. Therefore, it must be with caution when generalizing the present findings to NH residents with dementia who did not receive home-based care. Second, due to limited variables in the source of data, some major confounders, such as severity of dementia, laboratory data, characteristics of caregivers such as professional knowledge and attitudes to care, and the goals of NH care were unable to adjust in the analysis. These are important information that may impact the caregivers to make decision whether transfer the NH residents with dementia to the ED and are necessary to judge” care appropriateness” from clinical view. For example, unlike the NH residents with mild to moderate dementia, the care goals of NH residents with advanced dementia prefer care focused on comfort instead of aggressive treatments [52]. This condition may lead to the patterns of ED use different among these patients. Thus, residual confounding biases are inevitable when lack of these information in our study. Additionally, it is difficult to determine whether ED are overutilized or underutilized in the study. Considering that both overutilized and underutilized ED can have negative implications for patient health and health systems [53, 54], we suggest that factors associated with potentially overutilized or underutilized ED among NH residents with dementia should be further explored. Thus, research can provide references for preventing unnecessary or unmet cares. Third, the geographic location and the levels of urbanization in Taiwan were identified from the Taiwan NHI insured’s data, which may be not the actual place of residence of the insured. Thus, this residence misclassification may reduce the association between reginal factors and the risk of all-cause and respiratory-cause of ED use.

## Conclusions

This one-year cohort study revealed that respiratory diseases, especially pneumonia, were the leading cause of ED visits among NH residents with dementia. The predictors for the risk of all-cause and respiratory-cause ED visits included predisposing factors (i.e., age and gender), enabling factors (i.e., regional variables), and need factors (i.e., prolonged ventilator dependence and comorbidity status). In light of this, we provide some suggestions for clinical and policy implementation. First, developing an intervention aimed at reduction the most common and preventable cause of ED (i.e., respiratory diseases, especially pneumonia) contributed from prolonged ventilator dependence and from COPD would play a crucial role in decreasing ED utilization. Second, to identify the accessibility and availability of medical resources according to the reginal differences and then improve the above-mentioned situations appropriately may be another viable strategy to decrease ED utilization.

## Data Availability

The data that support the findings of this study are available from the Health and Welfare Data Science Center, Ministry of Health and Welfare (HWDC, MOHW) but restrictions apply to the availability of these data, which were used under license for the current study, and so are not publicly available. Data are, however, available from the authors upon reasonable request and with permission of HWDC (https://dep.mohw.gov.tw/DOS/cp-5119-59201-113.html).

## Abbreviations

AG: Andersen-Gill
AHR: Adjusted hazard ratio
CI: Confidence interval
COPD: Chronic obstructive pulmonary disease
ED: Emergency department
HR: Hazard ratio
ICD-9-CM: International Classification of Diseases, Ninth Revision, Clinical Modification
ID: Incidence density
NH: Nursing home
NHI: National Health Insurance
NHIA: National Health Insurance Administration
NHIRD: National Health Insurance Research Database
SD: Standard deviations
